# City Avoidance in the Early Phase of Psychosis: A Neglected Domain of Assessment and a Potential Target for Recovery Strategies

**DOI:** 10.3389/fpsyt.2019.00342

**Published:** 2019-06-03

**Authors:** Philippe Conus, Lilith Abrahamyan Empson, Zoé Codeluppi, Philipp Sebastien Baumann, Ola Söderström, Dag Söderström, Philippe Golay

**Affiliations:** ^1^ Treatment and Early Intervention in Psychosis Program (TIPP), Service of General Psychiatry, Department of Psychiatry, Lausanne University Hospital (CHUV), Clinique de Cery, Prilly, Switzerland; ^2^ Institute of Geography, University of Neuchâtel, Neuchâtel, Switzerland; ^3^ ISPS-suisse, Lausanne, Switzerland

**Keywords:** psychosis, urbanicity, city, stress, recovery, treatment

## Abstract

**Background:** A considerable amount of research has explored the link between living in an urban environment during childhood and the increased risk to develop psychosis. However, the urban milieu is more than a risk factor as it is also a place for socialization and enrichment. The aims of the current study were to explore, in a large sample of early psychosis (EP) patients, their pattern of use of the city, their perception when exposed to various critical stressors, and their sensitivity to diverse forms of stimuli.

**Methods:** We sent a questionnaire (based on previous work conducted in a group of patients, including video-recorded walk-along in the city and a literature review) to 305 EP patients and to 220 medical students.

**Results:** Response rate in patients was low (38%). City avoidance and negative perceptions towards the urban environment increased in patients after onset of psychosis. Patients’ tendency to avoid city center correlates with both problematic social interactions and stimuli perceived as unpleasant. Patients seemed less likely to enjoy urban spaces considered as relaxing, suggesting a lower capacity to benefit from positive aspects of this environment.

**Conclusions:** The development of psychosis influences the way EP patients perceive the city and their capacity to feel at ease in the urban environment, leading to a high rate of city avoidance. Considering the possible influence of city avoidance on social relations and the recovery process, the development of strategies to help patients in this regard may have a significant effect on their recovery process.

## Introduction

An important body of literature suggests that growing up in an urban environment during childhood is a risk factor for the later development of psychosis (1–4). Although various authors have proposed hypotheses to explain this correlation, the mechanisms involved in this phenomenon are still unknown. This is a matter of concern, considering the very high proportion of the world population living and growing in an urban environment, and this domain evidently deserves more research effort.

The urban environment is however more than a risk factor: it is also a place of enrichment, through interpersonal interactions, access to cultural events, and globally through socialization. Within the city and psychosis nexus, researchers in psychiatry have so far neglected to study the way patients experience the urban environment, how they use it, and to what degree they manage to gain access to it. This failure to address this aspect of the problem and the absence of studies doing so through the eyes of patients are limitations that need to be overcome. Indeed, the exploration of patients’ experience of the city environment may contribute not only to generate hypotheses to explain why growing up in an environment is a risk factor for later development of psychosis; it could also increase our understanding of the phenomenon of social withdrawal often described in psychosis patients.

In recent publications (5, 6), we have reported on a study that we are currently conducting in the context of a collaboration between psychiatrists, psychologists, geographers, and linguists. Through a combination of approaches, mixing exploratory focus groups with case managers, psychiatrists, and psychologists, interviews with patients, video-recorded go-along with a sample of 10 early psychosis (EP) patients, and a semi-structured interview with 20 EP patients, we managed to proceed to a first exploration of this domain.

In a first paper (5), we studied the way EP patients experience being in the city, with the aim to identify places of stress. This “unpacking of the city” revealed three ways to relate to the city among patients. While a first group tended to avoid the city center altogether, a second group used the city exclusively at certain times of the day and a third group reported having no problem in relation to the urban environment. When exploring sources of stress, patients mentioned four elements: a) crowd density; b) excess of stimuli, mainly auditory (sensory overload); c) situations of unavoidable social interactions; and d) hindrance to mobility (either by physical obstacles or by traffic).

In a second paper (6), we identified some of the strategies EP patients adopt in order to face these stressful elements, such as establishing sensory bubbles (through headphones or being accompanied by a friend), creating niches and breaks in the city (developing trajectories including parks or churches for example), or carefully programming trajectories in the city.

Based on these elements and on data stemming from the literature, we developed a questionnaire exploring the way EP patients use the city and the nature of their experience while being in an urban milieu. The aims of the study were to explore, in a large sample of EP patients, their pattern of use of the city, their perception of the various critical stressors that we identified through the previous abovementioned studies, and their sensitivity to various forms of stimuli. In addition, we wanted to assess the impact of the emergence of psychosis on these elements and to compare patients with a control group regarding these issues.

## Methods

### Patients and Control Groups

Patients included in this study stem from a clinical EP cohort receiving treatment in a specialized EP program (TIPP: Treatment and early Intervention in Psychosis Program) implemented in Lausanne, Switzerland, in 2004 (7). Based on a case management model, this program provides 3 years of treatment to patients aged 18 to 35 who have developed a psychotic disorder and have not had more than 6 months of treatment, with routine outcome assessments every 6 months. Since its implementation in our catchment area of 350,000 inhabitants, the program had provided treatment to more than 400 patients at the time of the study. Clinical case managers recruited the patients who were still involved in the program at the time of the study, and the research team contacted the rest of the cohort first by mail, followed by a phone call 2 weeks later. We recruited controls among students completing their third year of medical studies at Lausanne University, Switzerland. The local ethics committee approved the research protocol and all subjects provided informed consent to participate to the study.

### Development of the Questionnaire

The development of the self-administered questionnaire followed a succession of stages. First, we conducted video-recorded go-along through the city of Lausanne, with a sample of 10 EP patients. Second, in order to explore their reactions to the immersion in the urban milieu, we visualized and analyzed the video of the go-along in their presence in a process of video elicitation during which we took note of elements of the urban milieu generating either a sense of stress or a sense of protection. After spontaneous evocation of relevant elements by the patient, we completed the exploration through a fine-grained analysis of the go-along video in their presence and through questions proposed by the researchers (at least one geographer and one psychiatrist). These video elicitation sessions were video-recorded and subsequently analyzed by the research team. Third, we constructed a list of items based on the main factors of stress or protection that stemmed from the abovementioned procedure. Fourth, we used this list to conduct semi-structured audio-recorded interviews with 20 EP patients, exploring the same issue of factors of stress and protection in the city, but without conducting walk-alongs. Fifth, the verbatim of the interviews underwent thematic analysis, and the extraction of the main themes as well as a comprehensive literature review (Abrahamyan Empson et al., in revision) guided the design of the questionnaire. Finally, we refined the content of the questionnaire in the interdisciplinary research team and benefited from critical comments from clinical case managers through the organization of focus groups.

The questionnaire (available in French upon request to the corresponding author) consists of three main parts. The first section gathers information on the sociodemographic characteristics (for example, place of birth, migrant status, residential mobility, level of education, and marital status) that seemed relevant, based on a systematic literature review conducted in the frame of the project (Abrahamyan Empson et al., in revision). The second section evaluates the rate of city attendance (frequency and duration) and their perception of various specific places of the city (rated from very unpleasant to very pleasant). The third part explores the sensory and interactional dimensions of city living (ranging from sensitivity to sensory stimulations, to reactions towards interactions with other persons through gaze for example). All items are rated according to five-point Likert scales, where a score of 1 would mean “very pleasant” while 5 would represent “very unpleasant.” The questionnaire given to patients included additional questions regarding the impact of the development of psychosis on each particular dimension, rated from “much worse” to “no change” after the onset of psychosis.

### Data Analysis

Comparisons between groups were performed with independent *t* tests for continuous variables and Mann–Whitney *U* tests for ordinal or highly skewed variables. For nominal variables, analyses were performed with Pearson’s chi-square tests or Fisher exact tests when appropriate. Differences between perceptions before and after illness onset were tested with a one-sample sign test using the neutral value (no change) as the null hypothesis. Predictors of city avoidance were evaluated with two linear stepwise regression models fitted separately in each group. Items related to relation with others, gaze, and unpleasant stimulus were entered as independent variables with city avoidance as the dependent variable. All statistical analyses were performed with IBM-SPSS 23. All statistical tests were two-tailed and significance was determined at the .05 level.

## Results

Although 400 patients were eligible at the time of the study, the questionnaire was sent to 305 of them. Ninety-five questionnaires were not sent because of the following reasons: patient moved out of Switzerland, diagnosis of organic psychosis, substance abuse as first-line diagnosis, patients whose address could not be found, and patients who had died. While 124 questionnaires were returned, 117 (38%) were usable and 7 were incomplete. Among the 220 students who attended third year of medical school at the time of the study, 205 (93%) returned their questionnaire. The characteristics of both groups are shown in **Table 1**. Comparison of both groups revealed significant differences, with patients being significantly older than controls, and more likely to be male, to be a migrant, and to live independently. In addition, patients had moved houses more frequently and were less likely to have a regular activity at the time of the study.

**Table 1:** Patients and controls’ profile.

	Patients, *N* = 117	Controls, *N* = 205	Statistic	*p* value
Age, M (SD)	29.67 (5.85)	24.51 (6.65)	*t*(320) = 6.979	<.001
Gender, % male (*n*)	69.2 (81)	59.0 (121)	χ^2^(1) = 3.319	.068
Migrant status, %, (*n*)	40.5 (47)	20.0 (41)	χ^2^(1) = 15.672	<.001
Activity, % (*n*) Full or part time or studies Medical leave Unemployed, disability pension	25.3 (21) 31.3 (26) 43.4 (36)	100.0 (205) 0.0 (0) 0.0 (0)	χ^2^(2) = 195.142	<.001
Living status, % (*n*) Independent household In couple In couple with children With family Shared flat Pension/care home Unsettled Other	29.9 (35) 11.1 (13) 8.5 (10) 21.4 (25) 6.8 (8) 12.8 (15) 1.7 (2) 7.7 (9)	13.7 (28) 7.8 (16) 3.9 (8) 49.3 (101) 22.4 (46) 0.0 (0) 0.5 (1) 2.4 (5)	f	<.001
Number of moves, M (SD)	3.54 (2.22)	2.36 (2.20)	*t*(315) = 4.544	<.001
Time spent out of home, % (*n*) Almost never Less than 1 h Between 1 and 4 h More than 4 h	7.9 (9) 20.2 (23) 31.6 (36) 40.4 (46)	1.0 (2) 2.0 (4) 4.9 (10) 92.1 (187)	*U* = 5534.0	<.001
Diagnostic, % (*n*) Schizophrenia Schizophreniform/brief Schizo-affective Major depression^a^ Bipolar disorder Other	66.3 (55) 10.8 (9) 8.4 (7) 3.6 (3) 2.4 (2) 8.4 (7)	−	−	−

f, Fisher exact test.

a with psychotic features.

### City Attendance and Perception of the Urban Milieu

Patients go significantly less to the city center than controls (*U* = 8702.500, *p* < .001) and report a significant decrease in their city attendance since the occurrence of the first psychotic episode (*Z* = −5.715, *p* < .001). In addition, patients perceive city center as significantly more unpleasant than controls (*U* = 9219.000, *p* < .001). Here, again, their perception of the city became significantly less favorable since illness onset (*Z* = −6.013, *p* < .001).

### Sensory and Interactional Dimensions of City Attendance

Perception of the crowd is negative for the majority of patients, but this rate of negative perception does not differ from the perception by controls (*U* = 11,149.000, *p* = .320). Perception of the crowd is reported as worse in patients after illness onset than before (*Z* = −4.596, *p* < .001).

A smaller proportion of patients feel indifferent to (meaning undisturbed by) others than controls [χ^2^(1) = 5.179, *p* = .023] and a higher proportion of patients feel ill at ease with eye contact in the city than controls [χ^2^(1) = 20.128, *p* < .001] (see **Table 2**). In addition, openness to contact decreases significantly in patients after illness onset (*Z* = −3.283, *p* < .001).

**Table 2:** Perception of the gaze of others.

	Patients, *N* = 117	Controls, *N* = 205	Statistic	*p* value
Eye contact is stressful, % (*n*)	17.1 (20)	3.4 (7)	χ^2^(1) = 18.017	<.001
The gaze of others is bothering, % (*n*)	18.8 (22)	11.3 (23)	χ^2^(1) = 3.497	.061
I feel judged by others, % (*n*)	21.4 (25)	11.3 (23)	χ^2^(1) = 5.956	.015
I feel observed by others, % (*n*)	17.1 (20)	12.7 (26)	χ^2^(1) = 1.145	.284
I feel that the others analyze me, % (*n*)	18.8 (22)	9.8 (20)	χ^2^(1) = 5.295	.021
I feel threatened, % (*n*)	6.0 (7)	0.5 (1)	χ^2^(1) = 9.231	.002
I feel inferior, % (*n*)	14.5 (17)	2.0 (4)	χ^2^(1) = 19.213	<.001
I feel vulnerable, % (*n*)	14.5 (17)	4.4 (9)	χ^2^(1) = 10.227	.001
I am indifferent to the gaze of others, % (*n*)	28.2 (33)	36.8 (75)	χ^2^(1) = 2.440	.118

**Perception of various urban spaces:** Patients dislike crowded places to a similar extent as controls, but enjoy relaxing places with less intensity than controls (cf. **Table 3**).

**Table 3:** Negative perception of various urban spaces.

	Patients, *N* = 117	Controls, *N* = 205	Statistic	*p* value
Downtown center, Mdn (IQR)	3.0 (1.0)	2.0 (1)	*U* = 10608.0	.156
Mall, Mdn (IQR)	3.0 (2.0)	3.0 (1.0)	*U* = 10903.5	.368
Metro station, Mdn (IQR)	3.0 (1.0)	3.0 (1.0)	*U* = 10761.5	.238
Ouchy (lake shore), Mdn (IQR)	2.0 (2.0)	1.0 (1.0)	*U* = 7278.5	<.001
Parks, Mdn (IQR)	2.0 (2.0)	1.0 (1.0)	*U* = 7429.0	<.001
Old city, Mdn (IQR)	2.0 (1.0)	2.0 (1.0)	*U* = 7513.5	<.001^a^

a Patients > Controls.

**Sensitivity to external stimulations in the urban space:** 26.8% of patients reported feeling “flooded” by stimuli (sensory overload) compared to only 10.2% for the controls [χ^2^(1) = 14.681, *p* < .001]. This phenomenon worsened significantly in patients after illness onset (*Z* = −4.571, *p* < .001).

Patients were more likely than controls to consider visual elements (their complexity and the excess of visual stimulation) as unpleasant [χ^2^(1) = 9.549, *p* = .002]. However, controls were more likely than patients to consider noise [χ^2^(1) = 4.303, *p* = .038] and smell [χ^2^(1) = 40.697, *p* < .001] as unpleasant. There was no difference in the perception of physical contact with others between patients and controls [χ^2^(1) = 1.036, *p* = .309].

### Correlates of City Avoidance

The perception of certain distinct stimuli as unpleasant was significantly more likely to occur in patients who avoided specific places in the city. Sensitivity to physical contact was more likely to occur in patients who avoid going to the city center and to go in metro stations. Sensitivity to noise was more likely to occur in patients who avoid metro stations, downtown center, malls, and the old part of the city. In contrast, patients who do not report any stimuli as unpleasant are more likely to enjoy the city (see **Table 4**).

**Table 4:** Correlation between stimuli perceived as unpleasant and likelihood to avoid certain urban places.

	Stimuli perceived as unpleasant (*p* value of difference <.05)				
	Noise	Contact	Smell	Visual	No stimuli perceived as unpleasant
Avoid city center		.001			
Enjoy city center					.009
					
Avoid metro	.007	.020		<.001	
Avoid downtown center	.030				
Avoid old town	.032				
Avoid mall	.046				
Avoid lake			.033		
Enjoy all places					.001

A higher degree of city avoidance was found in patients with absence of openness to contact (*U* = 1047.0, *p* = .002), who felt disturbance by proximity with others (*U* = 762.5, *p* = .025), and who reported uneasiness with eye contact (*U* = 825.0, *p* = .001).

Taken together, city avoidance within patients was predicted by uneasiness with physical contact (β = .255, *p* = .005) and absence of openness to contact (β = .217, *p* = .017). Overall, this model was able to explain 13.4% of the variance of city avoidance. Within controls, city avoidance was only predicted by uneasiness with physical contact (β = .158, *p* = .025), which explained only 2.5% of the variance.

## Discussion

There are three main findings in our study. First, the development of psychosis seems to influence city perception and the rate of avoidance of the city among patients. Second, patients’ tendency to avoid city center correlates with both problematic social interactions and stimuli perceived as unpleasant. Third, comparison between patients and controls reveals similarities regarding the type of urban space characterized as unpleasant, but patients seemed less likely to enjoy urban spaces considered as relaxing, suggesting a lower capacity to benefit from positive aspects of this environment.

Patients globally report that the onset of the illness induces an increase in city avoidance, a greater feeling of uneasiness with the crowd and towards eye contact, as well as a global higher sensitivity to stimuli. In addition, onset of psychosis correlates with a marked decrease in time spent outside of home and regarding openness to others. The fact that patients report a similar degree of negative perception of the crowd to controls as well as the feeling that this perception has become much worse since illness onset suggests that they may overestimate their status before the first episode. Nevertheless, our data strongly suggest that living in a city has become much more difficult for patients after psychosis onset. This globally suggests that emergence of psychosis restricts capacities to use urban space and to access the options it offers, due to various changes in the perception of the urban milieu. In turn, this may partly explain the withdrawal observed in patients, and it could therefore be useful to focus on this aspect through specific interventions, considering the potential self-perpetuating nature of city avoidance.

City avoidance is associated with two domains of difficulties. First, it is apparently linked to problematic social interactions, such as a decrease in openness to contact with others occurring after illness onset, and uneasiness with eye contact and proximity. Globally, these elements are probably linked to a mechanism of self-stigma, which may also constitute a target for psychological treatments through psychoeducation and work on self-esteem. Second, city avoidance is also linked to stimuli perceived as unpleasant, principally noise and physical contact. This may be explained by the phenomenon of aberrant salience (8), where all stimuli gain similar importance and contribute to the feeling of flooding, which is reported by a third of patients. There, again, some strategies might be developed to help patients cope with stimuli in order to regain some freedom.

Finally, we were not astonished that the comparison between patients and controls revealed that the former are more avoidant of the city and more disturbed by eye contact than the latter. It was more revealing to observe that while patients and controls are similar in their dislike of crowded places, patients were less likely to enjoy relaxing places such as the lakeshore or parks. This suggests that patients strongly experience the negative aspects of the city, but that they fail to benefit from the positive aspects of relaxing environment, a phenomenon that may stem from anhedonia and/or self-stigmatization. It is important to explore this issue in more depth, considering that recent studies have shown that exposure to natural features within the built environment may have a positive impact on mental well-being in a normal population (9). Conducting similar smartphone-based studies in patient samples would clarify if such a beneficial effect is also present after illness onset. In addition, while controls were more likely to report some aspects of the city as unpleasant (for example, they were more likely to perceive noise and smell as unpleasant), city avoidance was much less prevalent among them than among patients, which illustrates their greater capacity to cope with these perceptions and to access the city.

There are obvious limitations to this paper. First, it stems from a relatively small sample of patients and a low percentage of responses. Indeed, only 38% of patients responded to the questionnaire, which induces an important risk of non-response bias. Although this response rate is in keeping with surveys in metal health research (10), our results need replication in larger groups of patients with a similar profile. Second, the assessment of the impact of illness onset on city perception and city avoidance in patients is retrospective, and as mentioned above, this could bias the results through an overestimation of the way they felt before illness onset. A prospective study conducted among At Risk Mental State (ARMS) (11) subjects may overcome this issue, although we know that ARMS subjects can already display important functional impairment (12). Third, the control group is composed of medical students exclusively, a group that may not be representative of the general population. However, although medical students may, at first sight, look like a “super-healthy group,” various studies and review papers have recently shown that the prevalence of mental health issues among them is high and may even exceed the rate of the general population (13, 14), therefore limiting the impact of this potential bias. Fourth, while some patients who filled in the questionnaire were still in treatment and therefore would have been accessible for clinical assessment, a large number of subjects were not available for symptoms evaluation. Considering the potential impact of depressive, anxiety, as well as positive or negative symptoms on city avoidance, the absence of such assessment is a limitation of the study. However, in this paper, we explore city avoidance per se in a descriptive manner and the reasons subjects put forth to explain it but do not attempt to identify which illness dimensions may explain it, which may be the focus of later studies.

Despite these limitations, our study suggests that the development of psychosis has a great influence on the way EP patients perceive the city and on their capacity to feel at ease in the urban environment, leading to a high rate of city avoidance and decreased opportunity to interact with others. This aspect of the impact of the illness has not received the attention it deserves, although city avoidance can have a major influence on social relations and the recovery process. The development of strategies to help patients in this regard may have a significant effect on their recovery and therefore should be explored in more depth.

## Ethics Statement

The local ethics committee (Commission cantonale (VD) d'éthique de la recherche sur l'être humain) approved the research protocol and all subjects provided informed consent to participate to the study.

## Author Contributions

PC, LA, ZC, PB, OS, DS, and PG have contributed to the design of the study, the grant application, the realization of the various stages of the research project, and the design of the questionnaire used in this part of the study. LA, ZC, and PB conducted the invoice of the questionnaire and the gathering of data. PG conducted the data analysis. PC and LA drafted the first version of the paper and all other co-authors contributed to the finalization of the paper.

## Funding

This study was funded by the Swiss National Science Foundation (grant number CR13I1_153320).

## Conflict of Interest Statement

The authors declare that the research was conducted in the absence of any commercial or financial relationships that could be construed as a potential conflict of interest.
